# Mortality Rate and Years of Life Lost Due to Ischemic Heart Disease in Southern Iran between 2004 and 2019: A Population-Based Study

**DOI:** 10.34172/aim.28159

**Published:** 2024-09-01

**Authors:** Mohammad Hossein Sharifi, Alireza Mirahmadizadeh, Jafar Hassanzadeh, Hamed Bazrafshan Drissi, Habibollah Azarbakhsh

**Affiliations:** ^1^Research Center for Traditional Medicine and History of Medicine, Shiraz University of Medical Sciences, Shiraz, Iran; ^2^Non-Communicable Diseases Research Center, Shiraz University of Medical Sciences, Shiraz, Iran; ^3^Research Center for Health Sciences, Institute of Health, Department of Epidemiology, Shiraz University of Medical Sciences, Shiraz, Iran; ^4^Cardiovascular Research Center, Shiraz University of Medical Sciences, Shiraz, Iran; ^5^Epidemiology Department, Faculty of Health, Ahvaz Jundishapur University of Medical Sciences, Ahvaz, Iran

**Keywords:** Ischemic heart disease, Iran, Join point regression, Mortality rate, Years of life lost

## Abstract

**Background:**

Cardiovascular diseases (CVDs) account for one-third of all deaths worldwide.

**Methods:**

In this cross-sectional study, we extracted all death records from the Electronic Death Registration System categorized as ischemic heart disease (IHD) based on age, gender, and the year of death according to ICD-10 for this cross-sectional analysis. The Fars province is situated in southern Iran with a population of about 4 million. An analysis of years of life lost (YLL) resulting from premature death from IHD was conducted using the World Health Organization’s 2015 YLL framework. The trend of the YLL rates was investigated using joinpoint regression.

**Results:**

In the Fars province, IHD was the cause of 46969 deaths throughout a 16-year study period, (2004 to 2019). Among these, 26,503 (56.4%) were men. The crude death rates per 100000 population for men and women were 84.2 and 66.5, respectively. The total YLL due to premature death due to IHD, during the 16-year study period, was 287625 in male, 209665 in female. The joinpoint regression showed a declining trend in the YLL rate associated with premature death. Annual Percent Change (APC) was -0.6% (95% CI -6.9 to 6.1, *P*=0.851) for males and -1.5% (95% CI -5.2 to 2.2, *P*=0.418) for females.

**Conclusion:**

The trends of the standardized mortality rate, YLL, and crude mortality rate held steady throughout a 16-year period. Planning for comprehensive primary and secondary prevention and increasing public knowledge of IHD are necessary.

## Introduction

 Ischemic heart disease (IHD) is the most common cardiovascular condition that accounts for one-third of deaths worldwide.^1,2^ IHD has two clinical manifestations: myocardial infarction and ischemic cardiomyopathy.^2^ The main pathological mechanism causing IHD is atherosclerosis, an inflammatory arterial disease accompanied by accumulation of cholesterol and abnormal metabolic processes caused by a number of risk factors.^3^ IHD is a serious public health issue that accounted for the majority of years lost to premature mortality in Iran’s population in 2019.^4^ Additionally, a growing number of non-fatal IHD patients have a social, economic, and disease burden in the community due to chronic disabilities and a low quality of life.^1^

 Iran, a nation in transition, has dealt with communicable diseases and is currently facing new issues related to non-communicable diseases (NCDs).^5^ Cardiovascular diseases (CVDs) accounted for 20%–23% of the disease burden and 46% of all mortalities in Iran, making it the country’s first leading cause of death.^6^ Heart disease and stroke are the two primary causes of disability-adjusted life years (DALYs) in Iran. A previous study estimates that the 10-year CVD risk for men and women will rise by 19.9% and 32.2%, respectively, between 2000 and 2030.^7^ The rising incidence of CVD may be linked to socioeconomic and cultural changes, industrialization and urbanization, nutritional transitions, insufficient physical activity, rising life expectancy, and metabolic syndrome factors.^6^ Considering the predicted rise in IHD in most nations, it is critical to assess risk factors and develop primary prevention strategies tailored to each country’s culture and characteristics.^8^ A thorough understanding of the trend of IHD in Iran is required for supporting policymakers to estimate Iran’s NCDs health budget.

 The financial effects of IHD include hospital stays, therapies, revascularization procedures, clinic visits, emergency room visits, and prescription drug treatments and cause loss 1%‒1.5% of gross domestic product (GDP) and accounts for 10% of total healthcare expenditure in some developing country.^1,9^ According to Raghfar et al, the Iranian population’s financial burden from coronary heart disease is estimated to be between $4715 and $4908 billion, with angioplasty costs accounting for the majority (47%) and drug costs accounting for the minority (1.15%).^10^ According to a previous study, the mean hospitalization cost for patients with IHD in Iran was 586.42 ± 472.51 USD per patient and 103.64 ± 100.29 USD per day.^11^ As a result, a study of the trend of IHD could help policymakers in conducting strategic planning for primary and secondary prevention.

 To the best of our knowledge, there is insufficient data on the prevalence of IHD in the southern part of Iran, and the existing data typically comes from cohort studies with small sample sizes or short follow-up periods. The current study sought to investigate the 16-year trend in the mortality rate and YLL of IHD in the Fars Province, a large Iranian province. Planners for the health system may find this study useful in helping them pay attention to and concentrate on primary preventative interventions.

## Materials and Methods

 This cross-sectional study investigated mortality rates and years of life lost (YLL) as a result of IHD from 2004 to 2019. Physicians with training performed the analysis in Iran. First, the electronic death registration system of the Ministry of Health and Medical Education was used to compile death statistics. Next, the International Classification of Diseases (ICD) code and the country protocol were used to code the cause of death (12). Using ICD-10 (Code I20-I25), the age, gender, and year of death of each individual with IHD and its complications were connected to determine the primary cause of death.^12,13^ Duplicate records were excluded due to similarities in the father’s name, death date, and national number.

 The projected population of the Fars province was determined using basic data from population censuses held between 1996 and 2016 as well as health centers, while allowing for annual population increase. Standardization was based on the 2013 standard population of low- and middle-income nations.^14^

###  Statistics

 Frequency and percentage were used in the reporting of qualitative data. During the study years, age standardized rate (ASR) and crude mortality of IHD-related deaths were computed by year of death and sex. The chi-square test was used to assess the mortality trend during the duration of the investigation. *P *values below 0.05 were considered statistically significant. For data analysis, SPSS version 25 and Microsoft Excel 2016 were utilized.

 The World Health Organization standard life table was used to calculate the YLL, which was based on the following relationship: life expectancy at birth by age and sex, number of deaths related to IHD by age and sex^15,16^:

 YLL = N Ce (ra) / (β + r) 2 [e-(β + r) (L + a) [-(β + r) (L + a)-1] – e– (β + r) a [–(β + r) a-1]]

 N = number of deaths by age and gender,

 L = standard life expectancy for the deceased of a given age and sex,

 r = discount rate (default: 0.03),

 β = age-weighting constant (default: 0.04),

 C = modified adjustment constant for age-weights (default: 0.1658),

 a = age at death

 e = the mathematical constant (default: 2.71).

 The World Health Organization’s 2015 YLL template and Microsoft Excel 2016 were used to analyze the YLL linked to premature deaths from IHD.

 A log-linear model was used in joinpoint regression to assess the trend of YLL over a number of years. Joinpoint regression analysis assesses growth or decline within each period and illustrates how patterns change over time. The annual percent change (APC), which is dependent on the line segment’s slope and the average yearly percent change, describes the line segment that results between joinpoints.^15,17^ calculating the conventional APC is a common analysis method. The APC is computed by fitting a basic linear model in order to calculate and assess long-term trends.^15^ The APC from segmented analysis can be used to determine the nonlinearity of a trend when it is not continuous during the time period of interest. The Joinpoint Regression Program version 4.9.1.0 was utilized to conduct the trend joinpoint analysis.

 The Shiraz University of Medical Sciences (SUMS) Ethics Committee examined and approved the study’s protocol (code: IR.SUMS.REC.1399.772). This study was conducted in accordance with SUMS’s ethical standards in every way.

## Results

 During the 16-year study period from 2004‒2019, a total of 46,969 deaths occurred due to heart ischemic disease in the Fars province. Of these, 26 503 cases were (56.4%) males and 20 466 (43.6%) were females. The crude mortality rate in males and females was 84.2 and 66.5 per hundred thousand population, respectively in 2019 and the standardized mortality rate for males and females was 90.5 and 70.8 per 100 000 population in 2019, respectively (Table 1).

**Table 1:** Crude and Age-Standardized Mortality Rate (Per 100,000 Population) Stratified by Gender and Year in Fars (Iran) 2004‒2019

**Year**	**Number of Death**			**Crude Mortality Rate**			**Age-Standardized Mortality Rate (95% CI)**		
	**Male**	**Female**	**Total**	**Male**	**Female**	**Total**	**Male**	**Female**	**Total**
2004	1489	1085	2574	80.0	61.0	70.8	106.0 101.9‒110.0)	89.1 (85.5‒92.7)	97.8 (95.1‒100.5)
2005	1771	1328	3099	95.7	74.7	85.4	120.4 (116.0‒124.9)	100.8 (96.8‒104.8)	110.6 (107.5‒113.6)
2006	2009	1528	3537	108.6	84.7	96.8	136.0 (131.3‒140.7)	110.9 (106.6‒115.1)	123.5 (120.3‒126.6)
2007	2225	1630	3855	119.1	89.2	104.3	140.2 (135.2‒145.1)	110.6 (106.3‒114.9)	125.5 (122.2‒128.8)
2008	2044	1542	3586	108.3	83.3	95.9	120.9 (116.3‒125.6)	96.5 (92.4‒100.7)	108.7 (105.6‒111.8)
2009	2103	1598	3701	110.4	85.2	97.9	116.6 (111.9‒121.4)	92.6 (88.4‒96.8)	104.6 (101.4‒107.8)
2010	2000	1515	3515	104.0	79.8	91.9	106.3 (101.8‒110.9)	81.9 (77.9‒85.9)	93.9 (90.9‒96.9)
2011	1382	1184	2566	71.16	61.6	66.4	69.3 (65.6‒73.1)	60.5 (57.0‒64.0)	64.7 (62.1‒67.2)
2012	1400	1046	2446	71.1	53.8	62.5	69.5 (65.8‒73.3)	54.8 (51.5‒58.0)	62.1 (59.6‒64.5)
2013	1438	1171	2609	72.1	59.6	65.9	70.2 (66.4‒73.9)	56.1 (52.7‒59.5)	62.8 (60.3‒65.3)
2014	1536	1182	2718	76.0	59.5	67.8	72.9 (69.1‒76.7)	56.4 (53.0‒59.8)	64.5 (62.0‒67.1)
2015	1404	1150	2554	68.5	57.3	63.0	65.4 (61.8‒69.0)	53.1 (49.8‒56.4)	59.1 (56.6‒61.5)
2016	1112	829	1941	53.7	40.9	47.3	51.9 (48.8‒55.1)	37.4 (34.7‒40.2)	44.5 (42.4‒46.6)
2017	1213	1026	2239	58.3	50.6	54.5	54.1 (50.8‒57.4)	46.1 (43.0‒49.2)	50.0 (47.8‒52.3)
2018	1643	1293	2936	78.6	63.6	71.2	75.3 (71.5‒79.1)	56.2 (52.7‒59.6)	65.4 (62.8‒68.0)
2019	1734	1359	3093	82.4	66.5	74.6	76.9 (73.1‒80.8)	55.3 (51.7‒58.8)	65.7 (63.1‒68.4)
Total	26503	20466	46969	84.2	66.5	75.5	90.5 (89.5‒91.5)	70.8 (69.9‒71.7)	80.6 (79.9‒81.3)
*P*-Value	-	-	-	0.960	0.811	0.901	0.545	0.281	0.422

 During the sixteen years of the study period, the crude mortality rate and standardized mortality rate fluctuated. The greatest number of deaths in males and females pertained to the age groups of 65‒79 years and over 80 years, respectively.

 The total YLL due to premature death due to IHD, during the sixteen-year study period, was 287,625 (9.13 per 1000 persons) in males, 209 665 (6.81 per 1000 persons) in females and 497 290 (7.98 per 1000 persons) in both sexes (Tables 2 and 3). Across all age groups, heart ischemic disease had the highest and lowest YLL in both sexes in the age group of 65‒79 years and less than 20 years, respectively (Table 2).

**Table 2:** Years of Life Lost Due to Premature Mortality, Stratified by Gender and Year in Fars (Iran) 2004‒2019

**Variables**		**2004**	**2005**	**2006**	**2007**	**2008**	**2009**	**2010**	**2011**	**2012**	**2013**	**2014**	**2015**	**2016**	**2017**	**2018**	**2019**	**Total**
		**No. YLL**	**No. YLL**	**No. YLL**	**No. YLL**	**No. YLL**	**No. YLL**	**No. YLL**	**No. YLL**	**No. YLL**	**No. YLL**	**No. YLL**	**No. YLL**	**No. YLL**	**No. YLL**	**No. YLL**	**No. YLL**	**No. YLL**
0‒19	Women	59	466	345	381	292	233	0	58	2468	120	212	205	88	149	88	91	5255
	Men	113	288	409	403	372	232	288	316	3033	118	207	355	146	118	28	90	6516
20‒34	Women	377	236	730	676	480	920	1712	455	1045	319	454	211	184	292	319	134	8544
	Men	812	1158	1533	1133	1519	1257	1374	711	3267	525	995	626	654	391	497	542	16994
35‒49	Women	1083	1057	1630	1343	1424	1487	1393	781	940	632	1067	1116	568	604	557	789	16471
	Men	3062	3987	3837	3556	3240	3541	2695	1823	2617	2041	2778	2120	1952	2120	2147	2786	44302
50‒64	Women	3024	3485	4082	4346	4044	3869	3707	3228	2308	2169	3258	2737	1895	2347	2329	2299	49127
	Men	4265	5600	6820	7093	6853	6020	6602	4557	4097	4212	6229	5414	4298	3918	5689	5388	87055
65‒79	Women	6776	7574	8009	8332	6911	6426	5253	4120	3670	3996	3915	3821	2917	3532	4514	4415	84181
	Men	7912	7879	8510	9080	7282	7376	6026	4105	3450	4623	4033	3740	3381	3160	4785	4708	90050
+ 80	Women	1343	2163	2659	2955	3162	3774	3765	3016	2230	3206	2923	2831	2119	2629	3470	3842	46087
	Men	1206	1922	2343	3120	3216	3642	3785	2597	2113	2678	2645	2543	1779	2433	3122	3564	42708
Total	Women	12662	14981	17455	18033	16313	16709	15830	11658	12661	10442	11829	10921	7771	9553	11277	11570	209665
	Men	17370	20834	23452	24385	22482	22068	20770	14109	18577	14197	16887	14798	12210	12140	16268	17078	287625

**Table 3:** Years of Life Lost (YLL) Per 1000 Persons and YLL Trend by Gender and Age Groups in Fars (Iran) 2004‒2019

**Age Groups**	**YLL (years)**			**YLL Rate (Per 1000 Persons)**			**AAPC for YLL Trend**			**P for YLL Trend**		
	**Male**	**Female**	**Total**	**Male**	**Female**	**Total**	**Male**	**Female**	**Total**	**Male**	**Female**	**Total**
0‒19	6516	5255	11771	0.64	0.53	0.59	-7.4 (-17.5, 3.8)	-1.9 (-20.3, 20.6)	-5.6 (-14.9, 4.8)	0.169	0.843	0.257
20‒34	16994	8544	25538	1.72	0.87	1.30	-7.1 (-11.9, -2.1)	-5.9 (-15,4.1)	-7 (-11.8, -2)	0.010	0.165	0.010
35‒49	44302	16471	60773	7.07	2.68	4.90	-6.5 (-8.5, -4.4)	-8.3 (-11, -5.4)	-7 (-9, -4.9)	< 0.001	< 0.001	< 0.001
50‒64	87055	49127	136182	25.60	14.41	20.00	-6.1 (-8.3, -3.8)	-8.2 (-10.1, -6.3)	-4.6 (-10.8, 2)	< 0.001	< 0.001	0.495
65‒79	90050	84181	174231	65.89	61.55	63.72	-4.8 (-10.8, 1.6)	-5.4 (-10.9,0.4)	-4.7 (-10, 0.9)	0.352	0.222	0.191
+ 80	42708	46087	88795	100.11	120.37	109.69	1.6 (-5.5, 9.2)	1.6 (-5.8,9.6)	1.4 (-5.4, 8.6)	0.114	0.186	0.136
Total	287625	209665	497290	9.13	6.81	7.98	-0.6 (-6.9, 6.1)	-1.5 (-5.2, 2.2)	-1.4 (-5.9, 3.2)	0.851	0.418	0.542

###  YLL Trend Due to Heart Ischemic Disease

 The 16-year trend of the YLL lost rate due to premature mortality was constant, according to the joinpoint regression: the annual percent change (APC) was -0.6% (95% CI -6.9 to 6.1, *P* = 0.851) for males, -1.5% (95% CI -5.2 to 2.2, *P* = 0.418) for females, and -1.4% (95% CI-5.9 to 3.2, *P* = 0.542) for both genders.

**Figure 1:**
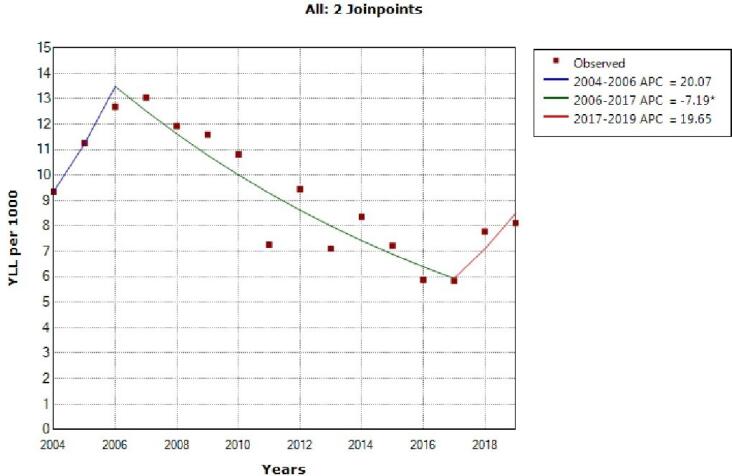
Trend of Years of Life Lost Due to Heart Ischemic Disease in Males During 2004‒2019. * indicates that the annual percent change (APC) is significantly different from zero at the alpha = 0.05 level. Final selected model: 2 joinpoints

 The model shows two joinpoint in 2006 and 2017 and 3 segment, 2004‒2006, APC was 20.1 (95% CI -18.5 to 77.7, *P* = 0.304), 2006‒2017, -7.2 (95% CI -10.0 to -4.4, *P* < 0.001) and 2017‒2019, 19.7 (95% CI -18.9 to 76.8, *P* = 0.317) for males (Figure 1).

**Figure 2:**
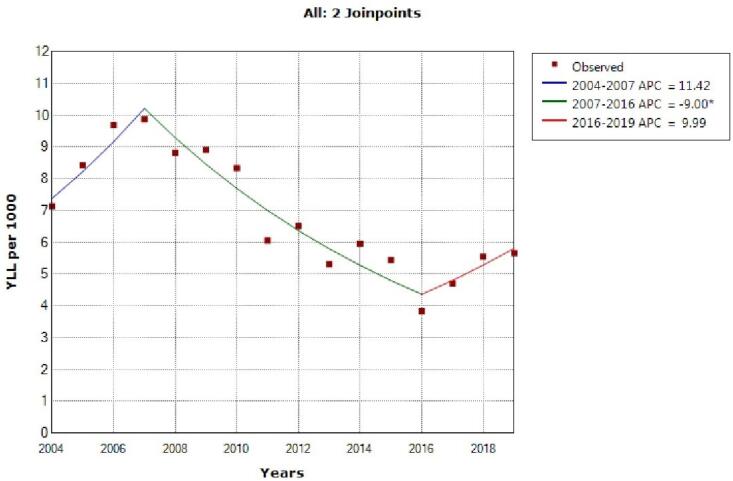
Trend of Years of Life Lost Due to Heart Ischemic Disease in Females During 2004-2019. * indicates that the annual percent change (APC) is significantly different from zero at the alpha = 0.05 level. Final selected model: 2 joinpoints

 The model shows two joinpoint in 2007 and 2016 and 3 segment, 2004‒2007, APC was 11.4 (95% CI -3.1 to 28.7, *P* = 0.111), 2007‒2016, -9.0 (95% CI -11.9 to -6.2, *P* < 0.001) and 2016‒2019, 10.0 (95% CI -4.4 to 27.0, *P* = 0.154) for females (Figure 2).

**Figure 3:**
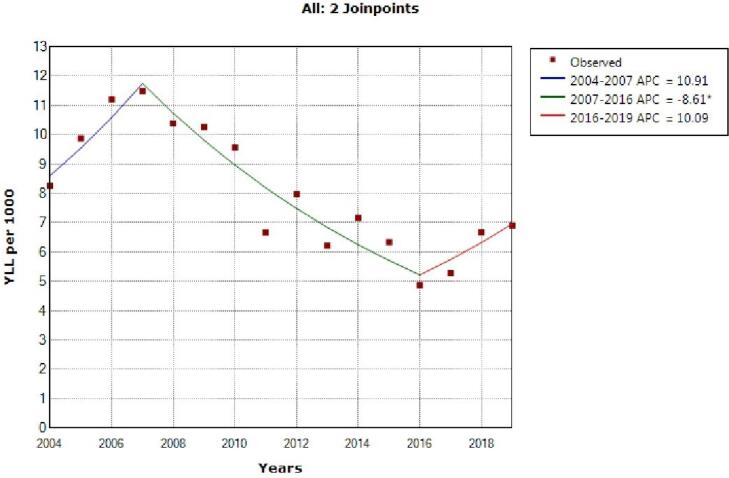
Trend of Years of Life Lost Due to Heart Ischemic Disease in Both Sexes During 2004‒2019. * indicates that the annual percent change (APC) is significantly different from zero at the alpha = 0.05 level. Final selected model: 2 joinpoints

 The model shows two joinpoint in 2007 and 2016 and 3 segment, 2004‒2007, APC was 10.9 (95% CI -6.8 to 31.9, *P* = 0.209), 2007‒2016, -8.6 (95% CI -12.0 to -5.1, *P* = 0.001) and 2016‒2019, 10.1 (95% CI -7.5 to 30.9, *P* = 0.240) for both sex (Figure 3).

 The trend of YLL due to heart ischemic disease showed a significant decrease in the age groups of 20‒34, 35‒49 and 50‒64 in males, and in the age groups of 35‒49 and 50‒64 in females (Table 3).

## Discussion

 In the last decade, little research has been conducted to determine the mortality and YLL associated with IHD in the Persian population. This study found that the crude mortality rate for males and females was (84.2 and 66.5 per 100 000 population, respectively), while the standardized mortality rate was (90.5 and 70.8 per 100 000 population, respectively). Furthermore, during the 16-year study period, the total YLL because of premature mortality due to IHD was 287 625 (9.13 per 1000 people) in men, 209 665 (6.81 per 1000 people) in women, and 497,290 (7.98 per 1000 people) in both sexes. The model indicates two joinpoints in 2007 and 2016, and three segments: 2004‒2007, APC was 10.8 (95% CI -6.8 to 31.9, *P*= 0.209), 2007‒2016, -8.6 (95% CI -12.0 to -5.1, *P*= 0.001), and 2016‒2019, 10.0 (95% CI -7.5 to 30.9, *P*= 0.240) for both sexes.

 IHD has been found to be the leading cause of death worldwide. Nevertheless, rates differ by country and have evolved over time. The mortality rates from IHD have significantly decreased in Western countries over the last few decades as a result of increased primary prevention efforts as well as improved IHD diagnosis and treatment.^2,18^ the regions with the greatest age-standardized death rates from IHD in 2019 were the Middle East, North Africa, and Eastern and Central Asia.^4,19^ the regions with the greatest age-standardized death rates from IHD in 2019 were the Middle East, North Africa, Eastern Europe, and Central Asia.^4,19^ It is noteworthy that there is insufficient detailed data on the present characteristics of the CVD epidemics in Asia.^20^ Our study’s findings show that, among the Iranian population, the standardized death rate was 70.8 and 90.5 per 100 000 people, while the basic mortality rate was 84.2 and 66.5 per 100 000 people. The crude and standardized mortality rates showed three segments trends throughout the course of the 16-year study period: an upward, downward, and upward trend. Furthermore, during the course of the 16-year study period, the total number of YLL lost to premature mortality from IHD was 287 625 (9.13 per 1000 persons) for men, 209 665 (6.81 per 1000 persons) for women, and 497 290 (7.98 per 1000 persons) for both sexes. The trend reveals two joinpoints in 2007 and 2016, and three segments: 2004‒2007, APC was 10.8 (95% CI -6.8 to 31.9, *P*= 0.209), 2007‒2016, -8.6 (95% CI -12.0 to -5.1, *P*= 0.001), and 2016‒2019, 10.0 (95% CI -7.5 to 30.9, *P*= 0.240) for both sexes. We consulted with scientists and officials who expertise in this area to obtain a better understanding and full explanation of this trend. In this respect, one explanation for this trend appears to be that, beginning in 2005, the adoption of primary PCI as the preferred therapy for patients with ST segment elevation, together with increased awareness and screening, resulted in a decrease in the number of deaths. Unfortunately, sanctions have been increasing since 2017. This could be attributed to Iran’s ongoing political problems. These issues have resulted in an insufficient number of expert doctors, the sanctioning and unavailability of the stent and equipment needed for PCI, and financial difficulties induced by the delay in patient referral. Other studies on IHD show a different trend. Each country appears to have a distinct tendency. Understanding the individual trend of IHD requires a full understanding of each country’s social, economic, and health-care systems.^17,21,22^

 According to our findings, the 65‒79 and above 80 age groups had the highest number of IHD deaths and YLLs. Another study discovered that the elderly have the most deaths and YLL lost due to IHD.^23-25^ the logical reason for these findings is that this age group has the highest prevalence of IHD and is most likely to develop disease-related consequences. Furthermore, older persons may have other health issues that increase the consequences of IHD, such as high blood pressure, diabetes, and obesity, increasing their risk of complications and death. In conclusion, due to a combination of factors, including age-related changes in the cardiovascular system, the presence of other health disorders, and diminished functional capacity, the aged population is particularly susceptible to the effects of IHD.^25^ Additionally, sarcopenia and atherosclerosis increase the risk of mortality and morbidity from IHD in this age range.^26^

 There are variations in the incidence, prevalence, and results of IHD by gender.^27^ Men have a higher incidence of IHD overall and are more likely than women to develop the disease at a younger age. However, after menopause, women’s risk of IHD rises dramatically, and the difference in occurrence between men and women shrinks.^27,28^ The current study also discovered that men had a higher YLL and crude and age-standardized death rate (per 100 000 population) than women over the course of the 16-year study period.

## Strengths and Limitations of This Study

 Some of the limitations of our study include the inconsistent quality of data collected throughout a 16-year period. In addition, other factors such as public awareness and screening initiatives, could have contributed to the 16-year difference. However, given that this is a population-based study with a sizable sample, policymakers may find it useful in assessing their programs and objectives. Future research ought to investigate the impact of numerous variables such as political changes, the number of skilled cardiologists, and health-care system support on the mortality rate and YLL due to IHD.

## Conclusion

 This study found that the crude mortality rate, standardized mortality rate, and total YLL increased with fluctuations throughout a 16-year period. Given the recent increase in the crude and standardized mortality rate and YLL due to IHD, it is critical for policymakers and decision-makers to raise awareness of IHD prevention, such as conducting priority planning to promote a healthy diet, regular exercise, availability of PCI equipment in hospital centers, and more trained cardiologists in hospitals. Dietary and physical activity modifications, as well as community-based health promotion programs, aimed at stopping alcohol and tobacco, lowering blood pressure, and lowering total cholesterol, have been shown to be successful interventions in various countries to reduce risk factors for IHD. Every nation should prioritize its research on developing the most effective preventative strategy. Future research to predict IHD in the next two decades could help health policymakers plan comprehensive primary and secondary prevention strategies.
